# Area-Level Disadvantage and Changes in Disability-Free Life Expectancy for Older Australians

**DOI:** 10.1093/geroni/igaa057.2198

**Published:** 2020-12-16

**Authors:** Richard Tawiah, Kaarin Anstey, Carol Jagger, Kim Kiely

**Affiliations:** 1 University of New South Wales, Bankstown, New South Wales, Australia; 2 University of New South Wales, Sydney, Australia; 3 Newcastle University, Newcastle upon Tyne, England, United Kingdom; 4 University of New South Wales, Sydney, New South Wales, Australia

## Abstract

We report the first analysis of inequalities in Disability-Free Life Expectancy (DFLE) trends for Australia, based on two cohorts of the nationally representative Household Income and Labour Dynamics in Australia survey. Each cohort was aged 45+ at baseline with 7-years of annual follow-up (Older cohort: 2001-2007, n=6363; Younger cohort: 2011-2017, n=8197). Disability was defined by a Global Activity Limitation Indicator, and socioeconomic position (SEP) by an area-level index of disadvantage. Compared to men in high advantage areas, men residing in low advantage areas experienced smaller gains in life expectancy (3.0 vs 4.6 years at age 65), DFLE (0.6 vs 1.8 years) and years with disability (2.4 vs 2.8 years). In contrast, for women in low advantage areas all years gained in life expectancy (2.6 years) were years with disability, whereas women in high advantage areas experienced gains in DFLE (1.7 years) and even more years with disability (2.7 years).

